# A randomised controlled trial of increasing fruit and vegetable intake and how this influences the carotenoid concentration and activities of PON-1 and LCAT in HDL from subjects with type 2 diabetes

**DOI:** 10.1186/1475-2840-13-16

**Published:** 2014-01-14

**Authors:** Jane-Ann Daniels, Ciara Mulligan, David McCance, Jayne V Woodside, Christopher Patterson, Ian S Young, Jane McEneny

**Affiliations:** 1 Centre for Public Health, Queen’s University Belfast, Pathology Building, Grosvenor Road, Belfast BT12 6BJ, UK; 2 Regional Centre for Endocrinology and Diabetes, Royal Victoria Hospital, Belfast BT12 6BA, UK

**Keywords:** Type-2 diabetes, Fruit and vegetables, High density lipoprotein, Carotenoids, Paraoxonase-1, Lecithin cholesterol acyltransferase

## Abstract

**Background:**

High density lipoproteins (HDL) have many cardioprotective roles; however, in subjects with type 2 diabetes (T2D) these cardioprotective properties are diminished. Conversely, increased fruit and vegetable (F&V) intake may reduce cardiovascular disease risk, although direct trial evidence of a mechanism by which this occurs in subjects with T2D is lacking. Therefore, the aim of this study was to examine if increased F&V consumption influenced the carotenoid content and enzymes associated with the antioxidant properties of HDL in subjects with T2D.

**Methods:**

Eighty obese subjects with T2D were randomised to a 1- or ≥6-portion/day F&V diet for 8-weeks. Fasting serum was collected pre- and post-intervention. HDL was subfractionated into HDL_2_ and HDL_3_ by rapid ultracentrifugation. Carotenoids were measured in serum, HDL_2_ and HDL_3_ by high performance liquid chromatography. The activity of paraoxonase-1 (PON-1) was measured in serum, HDL_2_ and HDL_3_ by a spectrophotometric assay, while the activity of lecithin cholesterol acyltransferase (LCAT) was measured in serum, HDL_2_ and HDL_3_ by a fluorometric assay.

**Results:**

In the ≥6- vs. 1-portion post-intervention comparisons, carotenoids increased in serum, HDL_2_ and particularly HDL_3_, (α-carotene, p = 0.008; β-cryptoxanthin, p = 0.042; lutein, p = 0.012; lycopene, p = 0.016), as did the activities of PON-1 and LCAT in HDL_3_ (p = 0.006 and 0.044, respectively).

**Conclusion:**

To our knowledge, this is the first study in subjects with T2D to demonstrate that increased F&V intake augmented the carotenoid content and influenced enzymes associated with the antioxidant properties of HDL. We suggest that these changes would enhance the cardioprotective properties of this lipoprotein.

**Clinical trial registration:**

ISRCTN21676269

## Background

Cardiovascular disease (CVD) is the leading cause of morbidity and mortality in Western societies, whose incidence is augmented by T2D [1]. In 2008, diabetes was the direct cause of 17% of all CVD related deaths [2]. Conversely, high density lipoproteins (HDL) are normally associated with reduced CVD risk, as these lipoproteins have many antiatherogenic properties; including their antioxidant and reverse cholesterol transport (RCT) capabilities. Furthermore, HDL stimulates glucose uptake and fatty acid oxidation, opposing insulin resistance [3]. However, in subjects with T2D the function of HDL may be defective, as hyperglycaemia increases lipid peroxidation [4], while the release of the inflammatory marker serum amyloid A (SAA) from hypertrophic adipocytes also impacts on the anti-inflammatory and antioxidant properties of HDL [5]. Thus the main enzymes associated with HDL’s antioxidant function, paraoxonase-1 (PON-1) and lecithin cholesterol acyltransferase (LCAT), with this latter enzyme also being involved in the maturation of HDL, may be altered to a pro-atherogenic phenotype in dysfunctional HDL [6,7].

Therefore, interventions that reduce the risk of diabetes and CVD, such as increased fruit and vegetable (F&V) intake, are highly desirable. Although the relationship between F&V intake and the incidence of T2D is not fully understood, there is substantial support for increased intake to reduce diabetes and CVD risk. In this regard, the study of Villegas et al. [8] reported that vegetables were protective against the development of T2D, while, in a population without diabetes, Panagiotakos et al. [9] identified that subjects consuming vegetables for more than 3 days per week had a 70% decreased risk of CVD and, furthermore, that an increase of one portion of fruit per day decreased CVD risk by 10%. In support of this, Cooper et al., [10] recently showed that increased vegetable intake and a variety of fruit reduced diabetes risk. Therefore, from the above evidence, it appears that increased F&V intake would be protective against the development of diabetes and the associated CVD complications.

The protective effect of F&V may be related to their rich antioxidant content, as these have been linked to a lower diabetes risk and the suppression of the inflammatory response related to diabetes and CVD development [11,12]. Many F&V derived lipid phase antioxidants, such as the carotenoids, are transported in the circulation by lipoproteins, including HDL, and in the case of lycopene, has been shown to influence the cardioprotective properties of HDL. In fact, in a previous study by our group we found that among middle-aged, moderately overweight subjects, lycopene intake, via supplement or as a lycopene-rich diet, lead to antiatherogenic increases in the activities of PON-1 and LCAT within HDL [11]. Additionally, another study identified that serum carotenoids and the activity of serum-PON-1 increased following a Mediterranean-like diet, while the inflammatory marker C-reactive protein (CRP) decreased [13]. Overall, these studies suggest that the antioxidants within F&V may, in part, be responsible for their cardioprotective effects.

However, although the literature is suggestive that increased F&V intake is beneficial to subjects with T2D, no study has investigated the relationship between increased F&V intake and the enzymes associated with HDL in these subjects. Therefore, the aim of this study was to examine if increased F&V intake had the potential to enhance the antiatherogenic properties of this lipoprotein in subjects with T2D.

## Methods

### Study population

**Figure 1:**
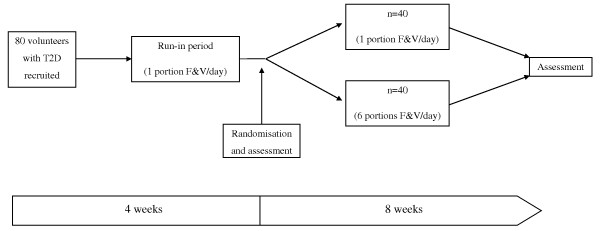
Study Design.

This study was a secondary analysis of blood samples collected from subjects with T2D following a randomised controlled F&V intervention. The study consisted of 80 subjects who were recruited from the diabetes outpatient clinics at the Regional Centre for Endocrinology and Diabetes at the Royal Victoria Hospital, Belfast, UK. All subjects were obese (BMI > 30 kg/m^2^), and aged between 40 and 70 years. In each case T2D was controlled by diet or oral hypoglycaemic therapy, which was maintained for the duration of the study. Prior to the 8-week intervention period all subjects underwent a 4-week run-in period, where they were asked to consume 1-portion of F&V/day. One portion of F&V is defined by the Food Standards Agency and the National Health Service as 80 g [14]. Following this, subjects were randomly assigned to 1 of 2 treatment groups (n = 40/group): a low F&V group, where they continued to consume 1-portion of F&V/day, or a high F&V group, were they consumed ≥6-portions of F&V/day. Fasting blood samples were obtained following the wash-out phase (week-0) and post-intervention (week-8), serum was isolated by centrifugation and stored in aliquots at -80°C until required for analysis. Figure 1 illustrates the study design for this intervention. F&V supplementation was similar to that of a normal diet, therefore, did not pose any health risk. Specific advice was also given to ensure a similar calorific and macronutrient intake to their normal diet, while weekly contact encouraged compliance. To assess compliance, two 7-day food diaries were completed during the run-in period and during the intervention (results not shown). Subjects selected the F&Vs of their choice, which were delivered weekly from a local supermarket during the 8-week intervention period. They were also offered advice on suitable storage and cooking methods for the F&Vs to minimise their degradation. This study was approved by the ethics committee of Queen’s University Belfast, and all subjects gave written informed consent.

### Exclusion criteria

Exclusion criteria included any acute coronary/cerebrovascular event, surgery within the previous 3-months; pregnant or lactating females; oral anticoagulant therapy; excess alcohol consumption (>2 units per day for females; >3 units per day for males); food sensitivities that would interfere with the F&V consumption; medical conditions or dietary restrictions that would substantially limit the ability of the subject to complete the study requirements; and ingestion of oral vitamin/antioxidant supplements in the 4-weeks prior to the wash-out phase.

### Isolation of HDL_2_ and HDL_3_ from serum

HDL_2_ and HDL_3_ were harvested from freshly thawed serum by rapid ultracentrifugation, according to the method of McPherson et al., (2007) [15]. This consisted of a 3-step, 6-hour long procedure. HDL samples were stored at -80°C and at this temperature HDL is stable for up to one year (results not shown), all samples were batch analysed.

### Apolipoprotein (apo) AI concentration

Apo AI was determined by a single radial immunodiffusion method, as previously described [16].

### Carotenoid concentration

Lipid soluble carotenoids, including α-carotene, β-carotene, β-cryptoxanthin, lutein, lycopene and zeaxanthin were measured in serum, HDL_2_ and HDL_3_ by reverse-phase-high performance lipid chromatography (HPLC) with diode array detection, as previously described [17].

### PON-1 activity

The arylesterase activity of PON-1 was measured in serum, HDL_2_ and HDL_3_. This assay was based upon the ability of PON-1 to hydrolyse phenylacetate, and was an adaption of the method of Hasselwander et al., 1999 [18], utilising 5 μL of serum, 200 μL of HDL_2_ and 20 μL of HDL_3_. One unit of arylesterase activity (U) is defined as 1 μmol of phenol generated per minute [18].

### LCAT activity

The activity of LCAT was measured in serum, HDL_2_ and HDL_3_ using a commercially available fluorometric assay (RB-LCAT, ROAR Biomedical, NY, USA), as per manufacturer’s instructions. The LCAT assay required 5 μL of serum, HDL_2_ or HDL_3_ per analysis.

### hsCRP and SAA concentration

The concentration of serum high sensitive (hs)CRP was determined by immunoturbidimetry. While SAA was measured in serum, HDL_2_ and HDL_3_ by a commercially available ELISA (Invitrogen, Human SAA KHA0011C) and the analysis was performed on a Grifols TRITURUS automated ELISA system (Italy), as per the manufacturer’s instructions, with the following modifications prior to analysis: serum was diluted 1:150, HDL_2_ 1:10 and HDL_3_ 1:100.

### Statistical analysis

A retrospective power calculation for HDL_3_-lycopene and HDL_3_-PON-1 activity indicated that a subject population of 20 per group was sufficient to give a 90% power at the 5% level of significance to detect a statistical difference between the groups. However, for completeness we have included all subjects in our analyses. Statistical analyses were performed using SPSS Statistics version 17.0. Variables were assessed for normality and logarithmically transformed where required. Between-group comparisons were analysed by an independent samples t-test. Within-group comparisons were analysed by a paired sample t-test. To adjust for potential confounders, an analysis of covariance was conducted, with final value of the variable of interest included as the dependent variable, the initial value of the variable of interest included as the covariate, and potential confounders, such as BMI, included in the model. All variables were summarised as mean (SEM) when normally distributed and as geometric mean (interquartile range) when normally distributed after logarithmic transformation. Correlations between variables were assessed by Pearson’s two-tailed bivariate analysis. Significance was set as p < 0.05 for all analyses.

## Results

### Subject characteristics

Initially 98 subjects were recruited into the study, 17 subjects withdrew during the run-in period prior to randomisation and 1 subject withdrew during the intervention period due to a family bereavement. A total of 80 subjects with T2D completed the 8-week intervention study. Six subjects had either pre- or post-intervention serum hsCRP levels >10 mg/l and thus were excluded from further analyses. This was in accordance with American Heart Association guidelines [19] as a hsCRP of >10 mg/l is taken as evidence of active infection or inflammation. Thus 74 subjects were included in the final analyses (Table 1). All baseline characteristics were comparable between the groups (p > 0.05 for all comparisons), and although there were more women randomised to the ≥6-portion intervention (n = 15/39) compared to the number of women randomised to the 1-portion intervention (n = 7/35), this was not statistically different (p = 0.295). Following intervention, BMI was different between the groups, which was driven by the significant increase in BMI (0.3 kg/m^2^) following the ≥6-portion intervention (p = 0.001). However, the effect of this change in BMI was tested independently using an analysis of covariance for all subsequent statistical analyses and was found not to influence the results (data not shown). Additionally, HbA_1C_ decreased following the 1-portion intervention (p = 0.032), although this was not continued in the between group comparisons (p > 0.05).

**Table 1:** Subject characteristics pre and post intervention

**Characteristic**	**1 Portion Group (n=35)**		**6 Portion Group (n=39)**		**Trend for change**
	**Pre**	**Post**	**Pre**	**Post**	**between groups**
**Male Sex (%)**	80	-	61.5	-	0.295
**BMI (kg/m**^**2**^**)**	30.1 (0.6)	30.1 (0.7)	30.7 (0.6)	31.0 (0.6)^d^	0.012
**Age (years)**	59.9 (1.23)	-	58.2 (1.47)	-	0.263
**Glucose (mmol/L)**	8.0 (6.6,9.6)	8.5 (7.5,22.3)	8.2 (7.0,8.9)	8.9 (8.3,18.8)	0.587
**Insulin (mU/L)**	13.7 (9.4,21.2)	14.6 (7.5,22.3)	11.9 (8.0,17.4)	16.8 (8.3,18.8)	0.470
**HbA1C (%)**	7.2 (0.2)	7.1 (0.2)^c^	7.1 (0.2)	7.1 (0.2)	0.068
**Duration of diabetes (years)**	6.7 (0.9)	-	5.2 (0.7)	-	>0.05
**Triglycerides (mmol/L)**	1.82 (1.2,2.1)	1.69 (1.1,2.1)	1.73 (1.1,2.0)	1.79 (1.2,2.2)	0.168
**Total cholesterol (mmol/L)**	3.95 (0.13)	3.86 (0.11)	3.87 (0.12)	3.93 (0.12)	0.640
**HDL cholesterol (mmol/L)**	1.24 (0.05)	1.20 (0.05)	1.25 (0.05)	1.23 (0.05)	0.194
**Lipid lowering therapy (%)**	94	-	95	-	>0.05
**Diabetic therapy (diet) (%)**	5	-	8	-	>0.05
**Oral hypoglycaemic therapy (%)**	86	-	80	-	>0.05

Results expressed as mean (SD) or when data was not normally distributed as geometric mean (interquartile range).

The following subscripts indicate - a; p≤0.05 for baseline comparison between groups: b; p≤0.001 for baseline comparison between groups: c: p≤0.05 for within group comparison: d; p≤0.001 for within group comparison.

### Fruit and vegetable consumption

F&V intake was calculated from two 7-day food diaries, where it was identified that F&V intake was significantly different in the ≥6- vs. 1-portion comparisons (p ≤ 0.001) following intervention, which was driven by the significant increase in F&V intake following the ≥6-portion intervention [pre 1.4 (0.5) portions F&V/day vs. post 5.2 (1.4) portions F&V/day, p ≤ 0.001], which equated to a 3.8 (1.34) portion increase in F&V/day, while F&V intake did not change following the 1-portion intervention [pre 1.2 (0.4) portions F&V/day vs. post 1.5 (0.9) portions F&V/day, p = 0.335].

### Dietary intake

Following intervention, total energy was not different in the ≥6- vs. 1-portion comparisons (p > 0.05; Table 2). However, carbohydrate and total sugar were significantly different between the groups (p < 0.05 and p ≤ 0.001, respectively), which were driven by the significant increase following the ≥6-portion intervention (p < 0.05 and p ≤ 0.001, respectively).

**Table 2:** Total energy intake and macronutrient intake pre and post F&V intervention

	**1 Portion (n = 35)**		**6 Portions (n = 39)**		**Trend for change**
	**Pre**	**Post**	**Pre**	**Post**	**between groups**
**Energy (kcal/day)**	1601 (370)	1646 (424)	1652 (494)	1748 (486)	>0.05
**Carbohydrate (g/day)**	181 (29)	186 (25)	184 (25)	215 (25)^d^	<0.05
**Total sugars (g/day)**	58 (28)	61 (22)	57 (17)	99 (25)^e^	≤0.001

Results expressed as mean (SD) or when data was not normally distributed as geometric mean (interquartile range).

The following subscripts indicate - a; p≤0.05 for baseline comparison between groups: b; p≤0.001 for baseline comparison between groups: c: p≤0.05 for within group comparison: d; p≤0.001 for within group comparison.

### Apo AI concentration

Following intervention, apo AI was not different in the between or within group comparisons (p > 0.05 for all comparisons, results not shown).

### Carotenoid concentration

Following intervention, serum α-carotene, β-cryptoxanthin, lutein and zeaxanthin increased in the ≥6- vs. 1-portion comparisons (p < 0.05 for all comparisons); Table 3) which were driven by increases of ~8% in α-carotene (p = 0.036), ~25% β-cryptoxanthin (p = 0.001) and ~25% lutein (p = 0.003), and the borderline non-significant increase in zeaxanthin (p = 0.067) following the ≥6-portion intervention. In HDL_2_, β-cryptoxanthin increased in the ≥6- vs. 1-portion post-intervention comparisons (p < 0.05), which was driven by the ~47% increase in β-cryptoxanthin (p = 0.015) following the ≥6-portion intervention, additionally, lutein also increased in HDL_2_ following the ≥6-portion intervention (p ≤ 0.001). For HDL_3_, α-carotene, β-cryptoxanthin, lutein and lycopene increased in the ≥6- vs. 1-portion post-intervention comparisons (p < 0.05 for all comparisons), which were driven by an increase of ~38% α-carotene (p = 0.001), ~32% β-cryptoxanthin (p = 0.041), ~9% lutein (p = 0.006) and ~27% lycopene (p = 0.015) following the ≥6-portion intervention. Additionally, following the ≥6-portion intervention there was a ~30% increase in β-carotene (p = 0.012) and a ~11% increase in zeaxanthin (p = 0.004).

**Table 3:** **Serum, HDL**_**2 **_**and HDL**_**3 **_**carotenoid antioxidants pre and post F&V intervention**

**Antioxidant**	**1 Portion (n=35)**		**6 Portions (n=39)**		**Trend for change**
	**Pre**	**Post**	**Pre**	**Post**	**between groups**
**Serum (μmol/L)**					
**α-carotene**	0.028 (0.004)	0.028 (0.005)	0.029 (0.003)	0.032 (0.004)^c^	0.047
**β-carotene**	0.10 (0.03)	0.11 (0.04)	0.13 (0.02)	0.14 (0.02)	0.520
**β-cryptoxanthin**	0.03 (0.01)	0.03 (0.01)	0.04 (0.005)	0.05 (0.005)^d^	0.002
**Lutein**	0.12 (0.01)	0.12 (0.01)	0.12 (0.01)	0.15 (0.01)^c^	0.030
**Lycopene**	0.28 (0.04)	0.31 (0.05)	0.35 (0.05)	0.34 (0.04)	0.820
**Zeaxanthin**	0.03 (0.003)	0.03 (0.003)	0.03 (0.003)	0.04 (0.002)	0.020
**HDL**_**2 **_**(nmol/L)**					
**α-carotene**	2.3 (0.3)	2.0 (0.4)	2.8 (0.3)	3.3 (0.7)	0.351
**β-carotene**	5.5 (1.1)	6.3 (1.5)	7.0 (1.1)	7.8 (0.1)	0.971
**β-cryptoxanthin**	2.0 (0.5)	1.7 (0.5)	1.9 (0.4)	2.8 (0.5)^c^	0.006
**Lutein**	5.5 (0.9)	5.9 (0.9)	5.9 (0.6)	7.6 (0.7)^c^	0.182
**Lycopene**	12.1 (1.2)	10.0 (1.1)	13.6 (1.3)	12.9 (1.2)	0.914
**Zeaxanthin**	3.1 (0.5)	2.8 (0.5)	2.9 (0.5)	2.7 (0.3)	0.826
**HDL**_**3 **_**(nmol/L)**					
**α-carotene**	8.1 (1.2)	8.5 (1.2)	7.8 (1.1)	10.8 (1.7)^d^	0.008
**β-carotene**	23.4 (3.2)	25.9 (4.5)	19.8 (4.3)	25.7 (5.5)^c^	0.399
**β-cryptoxanthin**	14.1 (2.4)	13.8 (1.9)	13.3 (3.1)	17.6 (2.9)^c^	0.042
**Lutein**	6.3 (0.7)	6.5 (0.7)	6.6 (0.4)	7.2 (0.4)^c^	0.012
**Lycopene**	33.1 (4.3)	30.3 (3.5)	29.3 (4.1)	37.2 (4.8)^c^	0.016
**Zeaxanthin**	10.5 (0.9)	10.7 (0.9)	9.7 (1.1)	10.8 (1.2)^c^	0.166
					

Results expressed as mean (SEM) or when data was not normally distributed as geometric mean (interquartile range).

The following subscripts indicate - a; p≤0.05 for baseline comparison between groups: b; p≤0.001 for baseline comparison between groups: c; p≤0.05 for within group comparison: d; p≤0.001 for within group comparison.

### PON-1 activity

Following intervention, serum-PON-1 activity was unchanged in the ≥6- vs. 1-portion comparisons (p > 0.05; Table 4), however, serum-PON-1 activity increased by ~7% (p ≥ 0.001) following the ≥6-portion intervention. In HDL_2_, PON-1 activity was unchanged in the ≥6- vs. 1-portion comparisons (p < 0.05) and following the 1 and ≥6-portion interventions (p > 0.05 for both comparisons). However, in HDL_3_, the activity of PON-1 significantly increased in the ≥6- vs. 1-portion comparison (p < 0.05), which was driven by the ~13% increase in its activity following the ≥6-portion intervention (p ≤ 0.001), despite the ~4% increase in its activity following the 1-portion intervention (p = 0.014).

**Table 4:** **Serum, HDL**_**2 **_**and HDL**_**3 **_**PON-1 and LCAT activities and SAA and hsCRP concentrations pre and post F&V intervention**

**Analyate**	**1 Portion (n=35)**		**6 Portions (n=39)**		**Trend for change**
	**Pre**	**Post**	**Pre**	**Post**	**between groups**
**Serum**					
**PON-1 (U/mL)**	25.78 (0.78)^a^	25.75 (0.78)	26.53 (0.81)	28.05 (0.79)^d^	0.155
**LCAT ratio (470/390)**	0.98 (0.04)	1.02 (0.03)	0.90 (0.02)	0.97 (0.02)^c^	0.562
**SAA** (**μg/L)**	21180 (6876,22894)	20926 (8898,22894)	21484 (6810,28645)	20464 (9020,31635)	0.399
**hsCRP (mg/L)**	2.30 (1.06,2.96)	2.19 (0.98,2.33)	2.17 (0.97,2.70)	2.34 (1.22,3.47)	0.527
**HDL**_**2**_					
**PON-1 (U/mL)**	0.37 (0.03)	0.38 (0.04)	0.40 (0.04)	0.42 (0.04)	0.959
**LCAT ratio (390/470) x10**^**-3**^	5.25 (0.05)	5.26 (0.06)	5.31 (0.07)	5.34 (0.08)	0.996
**SAA (μg/L)**	1579 (214,1188)	1448 (242,1107)	1145 (204,1120)	1197 (254,1196)	0.228
**HDL**_**3**_					
**PON-1 (U/mL)**	8.10 (0.21)	8.40 (0.20)^c^	8.54 (0.25)	9.51 (0.13)^d^	0.006
**LCAT ratio (390/470)**	0.89 (0.02)	0.90 (0.02)	0.87 (0.01)	0.94 (0.02)^d^	0.044
**SAA (μg/L)**	15834 (7549,21352)	17858 (5236,23360)	16211 (5007,24087)	12235 (4006,18120)	0.197

Results expressed as mean (SEM) or when data was not normally distributed as geometric mean (interquartile range).

PON-1 – paroxonase-1: LCAT – lecithin cholesterol acyltransferase: SAA – serum amyloid A

LCAT activity was read fluorometrically at 390 nm and 470 nm and expressed as a ratio of the two. These two wavelengths represent the LCAT substrate hydrolysed and not hydrolysed. An increase in the ratio indicates increased LCAT activity.

The following subscripts indicate - a; p≤0.05 for baseline comparison between groups: b; p≤0.001 for baseline comparison between groups: c; p≤0.05 for within group comparison; ^d;^ p≤0.001 for within group comparison.

### LCAT activity

In serum, LCAT activity was unchanged in the ≥6- vs. 1-portion comparisons (p > 0.05; Table 4). However, similarly to PON-1, the activity of serum-LCAT increased by ~8% following the ≥6-portion intervention (p = 0.025). In HDL_2_, the activity of LCAT was unchanged in the ≥6- vs. 1-portion comparisons (p > 0.05) and following the 1 and ≥6-portion interventions (p > 0.05 for both comparisons). However, the activity of LCAT was increased in HDL_3_ in the ≥6- vs. 1-portion comparison (p < 0.05), which was driven by the ~8% increase in its activity following the ≥6-portion intervention (p < 0.001).

### hsCRP and SAA concentration

Following intervention, serum hsCRP and serum-, HDL_2_- and HDL_3_-SAA were similar in the ≥6- vs. 1-portion comparisons (p < 0.05 for all comparisons; Table 4) and although SAA decreased by ~32% following the ≥6-portion intervention in HDL_3_, this was not significant (p = 0.133).

### Correlations between change in F&V intake, change in carotenoid antioxidant levels and change in activity of HDL-associated enzymes

Change in F&V intake was positively correlated with change in HDL_2_-β-cryptoxanthin (r = 0.312, p = 0.047), HDL_3_ α-carotene (r = 0.416, p = 0.005), HDL_3_ β-cryptoxanthin (r = 0.325, p = 0.032) and HDL_3_ lutein (r = 0.317, p = 0.036). Additionally, change in HDL_3_ β-cryptoxanthin positively correlated with change in HDL_3_-PON-1 activity (r = 0.258, p = 0.043).

## Discussion

### Carotenoids and the influence of increased F&V intake

This study has demonstrated that increased F&V intake augmented serum carotenoid levels in this subject population, illustrating subject compliance and confirming previous reports in healthy and T2D subjects [20]. In addition, we have shown for the first time in a population with T2D that this increase led to a concomitant increase in the carotenoid content of HDL_2_ and HDL_3_, which was particularly apparent for α-carotene, β-cryptoxanthin, lutein and lycopene in HDL_3._ Furthermore, this lycopene result confirms our previous findings, where we identified that lycopene, which is the most potent single oxygen quencher among the natural carotenoids [21], increased in HDL_2_ and HDL_3_ following a lycopene rich diet [11]. Thus we suggest that the increase in the carotenoids identified in this current study would enhance the antioxidant properties of HDL, similarly to that reported in subjects with T2D following a high polyphenol diet, where plasma malondialdehyde, a frequently used indicator of lipid peroxidation [22] decreased in subjects consuming this diet [23]. Furthermore, in support of our findings phylloquinone, a lipid-soluble molecule that is found in green leafy plants, improves markers related to insulin resistance and diabetes [24] and, due to its ability to accept electrons, contributes to the antioxidant capacity of HDL, which we suggest may also be the case for the increased carotenoids identified in this current study. In addition, a Mediterranean-style diet has been suggested to be advantageous from a metabolic perspective in subjects with T2D [25] and may even modulate genetic predisposition in such subjects [26]. Thus, overall we suggest that the increased antioxidants within the HDL subfractions, particularly HDL_3_, the subfraction more closely associated with HDL’s antioxidant properties [27,28], would have enhanced antiatherogenic potential in this T2D cohort.

### PON-1 and LCAT activities and the influence of increased F&V intake

This F&V intervention was also associated with significant increases in the activities of both PON-1 and LCAT, again confirming our previous findings following a lycopene rich diet [11]. Therefore, as one of HDL’s main antiatherogenic function is to inactivate peroxidised lipids removed from apolipoprotein B-containing lipoproteins, due to the proton donating properties of PON-1 [29] and to a lesser extent LCAT [30], an increase in the activities of these enzymes would be thought of as antiatherogenic. Therefore, as the activity of serum-PON-1 has been reported to be lower in subjects with T2D [31], we have now shown that increased F&V intake augmented its activity, in serum and particularly in HDL_3_, in this T2D population, which was further supported by the positive correlation between the concentration of β-cryptoxanthin and the activity of PON-1 in HDL_3_ (r = 0.258, p = 0.043). These findings are in support of previous studies, where quercetin and glabridin were shown to protect PON-1 against copper-induced oxidation [29], while pomegranate juice (a rich source of carotenoids) was shown to increase its activity in healthy male subjects [32]. Overall, we suggest that the increase in the described antioxidants may have enhanced PON-1’s proton donating properties and hence were responsible for the increase in its activity, especially in HDL_3_. We suggest that this increase in PON-1 activity would enhance the antiatherogenic potential of HDL in this T2D population.

With regards to LCAT, similarly to PON-1, this enzyme is mainly associated with HDL_3_, and its antioxidant property is related to it proton donating property [15,33]. Therefore, as the activity of LCAT has previously been reported to be decreased in subjects with T2D [34], we have now shown that increased F&V intake augmented its activity within serum and particularly in HDL_3_ in this T2D population. This increase in LCAT’s activity may be related to the proton donating capacities of the additional F&V derived antioxidants, thus illustrating another antiatherogenic effect of increased F&V intake.

### BMI, inflammation and the role of increased F&V intake

We must address the fact that BMI increased in the subjects randomised to the ≥6-portion intervention, in spite of the diets being designed and appearing isocalorific in both groups (p > 005). However, as both carbohydrate and total sugars significantly increased following the ≥6 portion intervention, and as these compounds are readily converted to fatty acids, this may have contributed to the increased BMI in this group, although a point to note is that these increases did not appear to influence the long term marker of glycaemic control, namely HbA_1C_, which was not different in the between group comparisons. Unfortunately, this increase in BMI may have negated a decrease in SAA, as there was only a trend for this inflammatory marker to decrease in HDL_3_ following the ≥6-portion intervention (p = 0.133). This was not consistent with previous non-reported findings by our group, where SAA did decrease following increased F&V intake, although in this study BMI remained stable. Therefore, the increase in BMI (0.3 Kg/m^2^) in the ≥6-portion group may have augmented SAA release, especially as SAA is released chronically from hypertrophic adipocytes [5]. Thus, the overall effect would be that any SAA-lowering effects afforded by the increased F&V intake would have been opposed by this additional adipose tissue. Overall, this finding raises a note of caution regarding the need to ensure that the composition of a F&V intervention should be closely monitored, to ensure that BMI remains stable. Nevertheless, this increase in BMI did not appear to impact on the enzymes examined in this current study.

Finally, we must consider the non-significant (p = 0.295) gender difference between the two groups at randomisation, where 20% of the subjects in the 1-portion intervention were female, while females made up 38.5% of the subjects in the ≥6-portion intervention. However, although females usually attach greater importance to healthy eating [35], all subjects appeared to adhere to the intervention group to which they were assigned, irrespective of gender. This was shown by the similar self-reported F&V intake and changes in carotenoids in both genders from each of the two groups (results not shown).

## Conclusion

In subjects with T2D, increased F&V consumption resulted in an increase in carotenoid levels and the activities of PON-1 and LCAT, which was particularly apparent in HDL_3_, which we suggest would augment the antioxidant capabilities of this lipoprotein and would enhance its antiatherogenic potential. This study provides mechanistic support for the effectiveness of increased F&V intake, which may be a realistic lifestyle intervention to help lower the risk of T2D and its associated CVD.

## Abbreviations

AI: (apo AI) Apolipoprotein; BMI: body mass index; CVD: Cardiovascular disease; F&V: Fruit and vegetable; FSA: Food Standards Agency; HDL: High-density lipoprotein; HPLC: High performance liquid chromatography; hsCRP: High-sensitivity C-reactive protein; LCAT: Lecithin cholesterol acyltransferase; PON-1: Paraoxonase-1; RCT: Reverse cholesterol transport; T2D: Type 2 diabetes.

## Competing interests

The authors declare that they have no competing interest.

## Authors’ contributions

JAD undertook the laboratory analyses for this study and drafted the manuscript. CM (formally McLaughlin) was responsible for the recruitment and running of the primary study and commented on the current manuscript. DMcC supervised CM in the primary analyses and also commented on the current manuscript. JW was involved in the primary analyses and also commented on the current manuscript. IY supervised CM in the primary analyses and also commented on the current manuscript. JMcE was responsible for the study concept, supervised JAD and also had the final say in manuscript preparation and submission. All authors read and approved the final manuscript.
